# Validation of Electroencephalographic Recordings Obtained with a Consumer-Grade, Single Dry Electrode, Low-Cost Device: A Comparative Study

**DOI:** 10.3390/s19122808

**Published:** 2019-06-23

**Authors:** Héctor Rieiro, Carolina Diaz-Piedra, José Miguel Morales, Andrés Catena, Samuel Romero, Joaquin Roca-Gonzalez, Luis J. Fuentes, Leandro L. Di Stasi

**Affiliations:** 1 Department of Signal Theory and Communications, University of Vigo, 36310 Vigo, Spain; hrieiro@ugr.es; 2 Mind, Brain, and Behavior Research Center, University of Granada, 18071 Granada, Spain; jmmorales@ugr.es (J.M.M.); acatena@ugr.es (A.C.); 3 College of Nursing and Health Innovation, Arizona State University, Phoenix, AZ 85004, USA; 4 Department of Computer Architecture and Technology, University of Granada, 18071 Granada, Spain; sromero@ugr.es; 5 Department of Bioengineering, Technical University of Cartagena, 30202 Cartagena, Spain; jroca.gonzalez@upct.es; 6 Department of Basic Psychology and Methodology, University of Murcia, 30100 Murcia, Spain; lfuentes@um.es

**Keywords:** brain activity, electroencephalography, driving simulator, low-cost wearables, NeuroSky^®^ MindWave Mobile headset

## Abstract

The functional validity of the signal obtained with low-cost electroencephalography (EEG) devices is still under debate. Here, we have conducted an in-depth comparison of the EEG-recordings obtained with a medical-grade golden-cup electrodes ambulatory device, the SOMNOwatch + EEG-6, vs those obtained with a consumer-grade, single dry electrode low-cost device, the NeuroSky MindWave, one of the most affordable devices currently available. We recorded EEG signals at Fp1 using the two different devices simultaneously on 21 participants who underwent two experimental phases: a 12-minute resting state task (alternating two cycles of closed/open eyes periods), followed by 60-minute virtual-driving task. We evaluated the EEG recording quality by comparing the similarity between the temporal data series, their spectra, their signal-to-noise ratio, the reliability of EEG measurements (comparing the closed eyes periods), as well as their blink detection rate. We found substantial agreement between signals: whereas, qualitatively, the NeuroSky MindWave presented higher levels of noise and a biphasic shape of blinks, the similarity metric indicated that signals from both recording devices were significantly correlated. While the NeuroSky MindWave was less reliable, both devices had a similar blink detection rate. Overall, the NeuroSky MindWave is noise-limited, but provides stable recordings even through long periods of time. Furthermore, its data would be of adequate quality compared to that of conventional wet electrode EEG devices, except for a potential calibration error and spectral differences at low frequencies.

## 1. Introduction

Electroencephalography (EEG), since its invention in the early 1900s [1], has been one of the most commonly used techniques for neurological and psychological assessments. Traditionally, EEG measurements have been performed with highly sensitive electronic devices in an attempt to maximize the signal-to-noise ratio, and using multiple electrodes (32, 64, 128, or more—usually reusable—embedded in a stretch-lycra electrode cap or pasted to the scalp). Typically, these expensive devices (with prices ranging from $5,000 to $50,000) restrict data collection to controlled laboratory environments, requiring participants to be physically tethered to them. Furthermore, they involve extensive training and experience for experimental setup and data collection [2,3].

Starting in the seventies, neuroscientists and neural/biomedical engineers have leveraged the potential of this technique in more applied settings, including brain computer interface (BCI) applications [4,5]. However, despite early interest to explore brain activity in more realistic contexts, for example to improve workplace safety [6] or to assess sleepiness during day and night work [7], EEG has only slowly gained traction in real-world settings [8,9], mainly due to the bulkiness and cost of the equipment. Nevertheless, in the past ten years new EEG devices [10] and processing algorithms [11] have appeared that overcome many of these barriers (for a recent review on this topic, see [12]). Their improved design offers simple arrangements that do not limit participants’ behavior and are easy to set up by researchers and general public, as they require little to no training [13,14,15]. Recent advances in dry electrodes technology have facilitated the recording of EEG in situations not previously possible [16,17]. Finally, their cost (from $99 to $500) has also become highly competitive, which makes these new EEG devices easily accessible to a wide commercial market, pushing BCI towards mass consumer adoption [18]. However, the functional validity of the EEG signal acquired with low-cost neurotechnologies is still under debate [19], and the quality (accuracy and reliability) of the data acquired with most of these low-cost EEG devices have not been fully proved yet [20].

The NeuroSky^®^ MindWave Mobile headset (NeuroSky Inc., San Jose, CA, USA, henceforth MindWave) is one of the most popular and affordable (about $99) low-cost EEG devices. Furthermore, the current adoption trend for this device [21,22,23,24,25,26,27,28] makes it imperative to help researchers and final users understand its validity. MindWave’s developers claim [29] it is able to measure cognitive functions, such as attentional and relaxation states, with only one passive dry electrode on the forehead, located at Fp1 (left frontal pole). However, quantitative studies of its actual validity for sensitively measuring EEG signals are limited to a manufacturer-provided white paper [29], the assessments carried out by Johnstone and colleagues of the previous version of this device [30,31], and another two works that compared the MindWave with wireless wearable EEG devices [32,33]. Differences in experimental methodology, such as the analysis of raw vs MindWave’s processed data, size of the study population (5 vs 20), as well as in recording techniques (simultaneously vs consecutively testing), make a direct comparison of these findings difficult [10]. Thus, results on the functional validity of the MindWave are not conclusive and the question of whether MindWave might be reliable enough to track overall EEG signal remains open (e.g., [32], but see [33]). 

Here, we carried out the first, in-depth study of the EEG recording quality of the MindWave device by performing simultaneous recordings with a medical-grade ambulatory electroencephalograph (SOMNOwatch + EEG-6, SOMNOmedics GmbH, Randersacker, Germany). Under well-controlled experimental conditions, we compared EEG signals acquired from virtually the same scalp place (Fp1 vs AF3) while participants performed laboratory tasks (e.g., a resting state task, alternating closed and open eyes). Furthermore, considering the growing interest for implementing tools to monitor cognitive performance in naturalistic environments [9], EEG signals were acquired also during a 1-hour long every-day activity (i.e., a simulated driving task).

The results presented here give an accurate representation of the strengths and limits of the MindWave recording device, and delineate the most appropriate scenarios for its use in scientific applications. 

## 2. Methods

### 2.1. Participants

In conformity with the Code of Ethics of the World Medical Association [34] and under the guidelines of the University of Granada’s Institutional Review Board (IRB approval #24/CEIH/2015), we recruited 21 active drivers (ten females) between the ages of 20 and 40 (mean age ± standard deviation [SD] = 25.14 ± 4.69 years). All participants were volunteers, had normal or corrected-to-normal vision, and held a valid driver’s license. We used medical history of significant head injury or neurological disorder as exclusion criteria. Furthermore, to reduce the influence of other potential confounder variables (e.g., participants not fully rested taking part in the study), we also considered low levels of arousal before the driving task (a score greater than 3 on the Stanford Sleepiness Scale, SSS [35]) as an exclusion criterion. No participants were excluded based on these criteria. 

### 2.2. Instruments and Materials

Neurosky^®^ MindWave (NeuroSky Inc., San Jose, CA, USA). This device consists of a single dry electrode (12 mm × 16 mm) placed on Fp1, according to the international 10-20 system [36], which inputs data to a TGAM1 (ThinkGear ASIC Module) integrated circuit. These two elements are mounted on a light headset (90 g). The device uses a monopolar montage with one active site, and employs a pea-sized (~0.8 mm diameter) electrode clipped to the left earlobe as reference. The device samples data at 512 Hz. The MindWave electrodes are made of stainless steel and all connections use shielded cables. Energy is supplied by a single 1.5 V AAA battery (for more details, see Table S1). The manufacturer has rated the device for continuous 8-hour operation on a single battery. Nevertheless, we took the precaution of changing the batteries after every 2 hours of use [37]. The headset uses a wireless Bluetooth connection to send EEG raw data to a recorder platform. We collected the raw EEG data into EDF+ (European Data Format) files using ad hoc LabVIEW (National Instruments Co., Austin, TX, USA) software.

SOMNOwatch + EEG-6 (SOMNOmedics GmbH, Randersacker, Germany). For a fair comparison between devices, we selected the SOMNOwatch + EEG-6 acquisition device (hereafter, SOMNOwatch). Both the MindWave and the SOMNOwatch devices are small, wearable devices, reasonably affordable, intended for applied (clinical or research) studies, and require a short setup time. The SOMNOwatch is generally used to perform ambulatory polysomnography [38] (i.e., to record EEG during sleep at home), and its reliability for sleep staging has been confirmed [39]. It has been used for research purposes as well (e.g., to record EEG in real-life settings [8,40]). Thus, it is robust to movements and noise, as well as artifacts from electrode movement that lead to changes in contact impedance, or even the generation of a triboelectric response on the wires.

**Figure 1:**
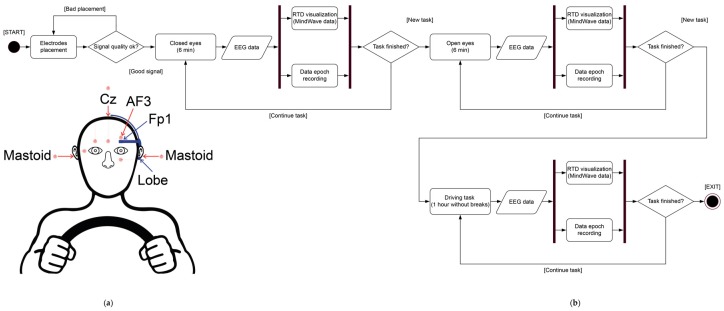
Experiment structure. (**a**) EEG recording configuration. Red elements and arrows indicate the electrodes used by the SOMNOwatch device. Blue elements and arrows indicate electrodes used by the MindWave device. (**b**) UML (Unified Modeling Language) activity diagram for the implementation and data acquisition of the experiment. The session started with the placement of the electrodes. After checking the signal quality, EEG data collection started. The experiment started with the resting state task (~15 min) structured as two cycles of task 1 (closed eyes, 3 min each) and task 2 (open eyes, 3 min each). The experiment started with either task 1 or task 2, as the order was random for each participant. Afterwards, the driving task (task 3) started (a 60-minute driving session without breaks). MindWave data was visualized in real time (RTD visualization) for all tasks. Once the three tasks finished, the session ended.

This device consists of two small thin boxes (SOMNOwatch and EEG headbox with ten wired electrodes) fastened to the chest with flexible belts. In this setup, it can record EEG, electrooculographic (EOG), and electromiographic data, as well as the position of the body. The device samples data at 256 Hz applying a band pass filter (0.1–80 Hz) (for more details, see Table S1). Impedance was kept below 5 kΩ for all electrodes. We used a monopolar montage with gold cup electrodes (Natus Neurology Incorporated—Grass Products Warwick, Pleasanton, CA, USA) at five active scalp sites: AF3 (right above Fp1), Fpz, C3, C4, and Cz (online reference) placed according to the international 10/20 system [36], and using the left mastoid (A1) as the offline reference. Ground was placed at Fp2. We analyzed the EEG activity of the channel AF3, which is the closest channel to Fp1 (localization of the MindWave electrode, see Figure 1). We recorded vertical and horizontal EOG from the outer canthus of the right eye and below the left eye using a bipolar configuration. The device collects internally the raw EEG data. We used the DOMINO Light software (version 14.0, SOMNOmedics GmbH, Randersacker, Germany) to export raw signals to EDF+ files.

Resting state EEG (no-task condition). We used a resting-state EEG experimental paradigm to analyze brain activity in the absence of any specific task. Thus, we designed a no-task closed eyes/open eyes resting state session: four periods of three minutes each in which participants alternated two closed eyes and two open eyes periods (12 min total). The first period (with the eyes open or closed) was randomly assigned to participants. The participants had to blink rapidly for 5 seconds to signal the start/end of both tasks (the whole eyes open/eyes closed task and the driving task). We used the blinks bouts as biological triggers. A researcher (author C.D.-P.), seating behind the participants, was in charge of giving participants instructions about the changes in periods. The resting state was always performed under no light/sound stimulation. Participants were asked to hold still with their hands resting on their legs and to direct their gaze toward infinity in the direction of a blank wall during the open eyes session.

Driving simulator task. We used a 60-minute driving session to analyze brain activity while participants were performing an ecological and dynamic task requiring controlled attention but not excessive mental effort [21]. We developed a two-lane rounded rectangle virtual circuit using the OpenDS 2.5 software (OpenDS, Saarbrücken, Germany). Participants, seating on a car seat (PlaySeat^®^, Almere, The Netherlands), drove a middle-sized car for one hour without breaks around the circuit in sunny conditions and without any other traffic present. The absence of traffic or intersections minimizes motion artifacts due to head movements, especially in a head-unrestrained condition. To control the car, participants used a Logitech G27 steering wheel (steering wheel with active dual-motor force feedback, gas and brake pedals; Logitech International S.A., Lausanne, Switzerland). Six loudspeakers located around the driver, at about ground level, provided the simulated surround sound of the engine. Speedometer and tachometer gauges were shown in the bottom right of the screen. A speed limit of 60 km/h was set. Each simulation included approximately thirty full laps around the simulated circuit (average number of laps ± SD = 32 ± 3); thus, all subjects saw/heard approximately the same visual/auditory stimuli during the task. We used a video projector (EB-410W, EPSON, Suwa, Japan) to display the virtual circuit on a 1.32 m × 1.63 m screen, about 2.5 m from the driver’s eyes (resulting in a view angle of ~30° vertically and ~36° horizontally). During the driving period, the projected image on the wall provided the only light inside the simulation laboratory.

### 2.3. Procedure

The experiment took place in a simulation laboratory (for more details see [41]), located at the Mind, Brain, and Behavior Research Center (Granada, Spain). First, the participant signed the informed consent form. We performed an initial screening to assess inclusion and exclusion criteria and to collect information about sociodemographic characteristics and driving experience. Then, while the participant was seating in a comfortable chair, the pertinent areas of skin were cleaned up with a slightly abrasive paste and alcohol before we placed the electrodes on his/her scalp. Gold electrodes were filled with conductive paste and pasted with collodion. Due to the instability of the MindWave EEG headset, the dry electrode was placed and secured with surgical tape to facilitate the adherence with the forehead skin. To reduce the impedance between skin and electrodes, to the extent possible, we ensured that hairs were put away [42]. Once participants were fitted with the devices and seated in the car seat, they filled in the SSS scale and drove during five minutes to familiarize themselves with the simulator. After that, they started the resting state EEG. Finally, the 60-minute driving simulation started. All participants were told to follow the usual traffic rules, such as keeping their speed below 60 km/h) and to keep the car in the right lane. 

### 2.4. Data Preprocessing

**Figure 2:**
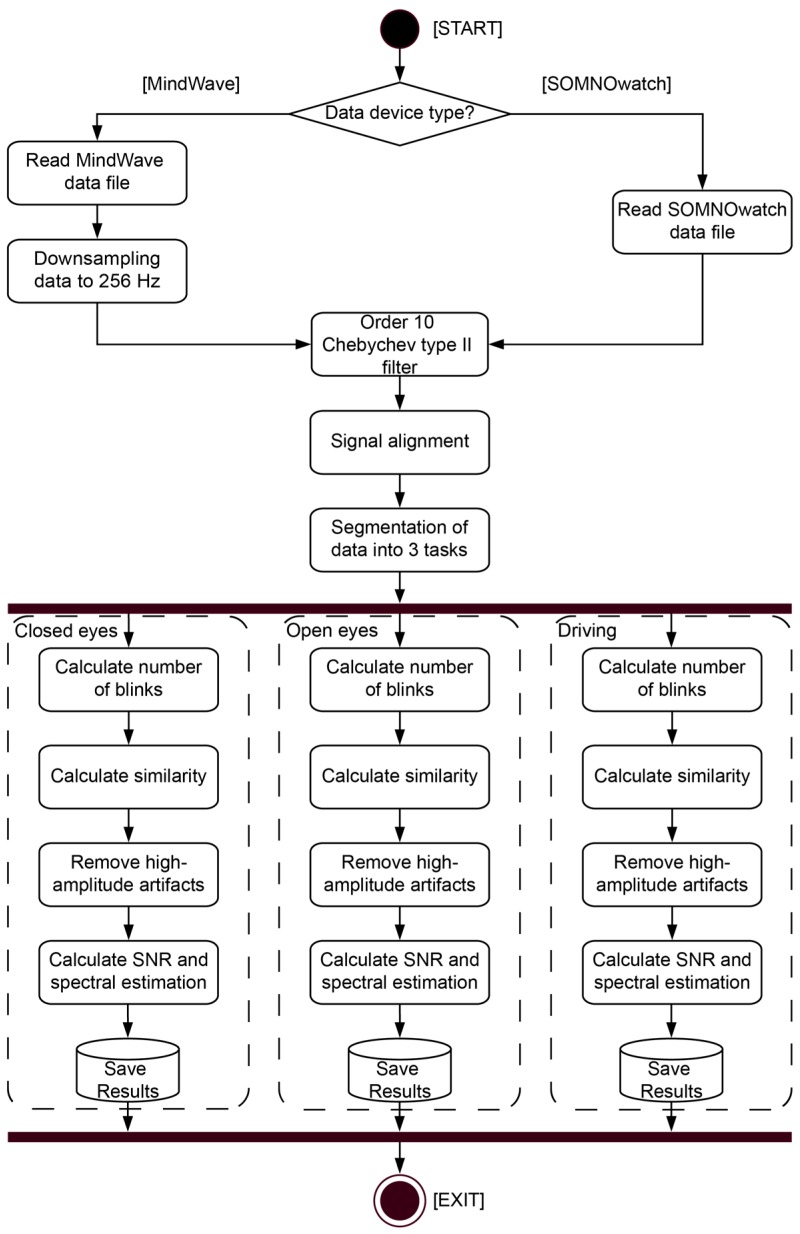
Data processing. UML (Unified Modeling Language) activity diagram for the experimental data processing. The process started by checking the device type. For the MindWave, we read the data file and downsampled it to 256 Hz; for the SOMNOwatch, we just read the data file (already at 256 Hz). Next, we applied an Order 10 Chebychev type II filter, followed by a signal alignment. We segmented the data into the three tasks (closed eyes, open eyes, and driving tasks). For each task, we detected blink artifacts, calculated the similarity measure, and removed high-amplitude artifacts, to finally compute the signal-to-noise ratio (SNR) and to perform the spectral estimation. Note that rectangles indicate processes, diamonds indicate decisions, and parallelograms indicate output data.

We imported, preprocessed, and analyzed EDF+ files using MATLAB (Mathworks Inc., Natick, MA, USA) (Figure 2). In order to facilitate the comparison between the waveforms of the recordings, we downsampled the MindWave signal from 512 Hz to 256 Hz (same as the SOMNOwatch device). Both signals were filtered using an order 10 Chebychev type II filter, which provides a sharp transition between passband and stopband without causing rippling in the former, to remove spectral components outside the [0.1 Hz, 45 Hz] interval. The recordings were aligned using an information-theoretic delay criterion [43]. We segmented the five periods including the two cycles of closed eyes and open eyes conditions (a 6-minute cycle), as well as the driving period (a 60-minute session).

Before analyzing the quality of the recording (signal-to-noise ratio [SNR] analysis and spectral estimation, see below), a threshold technique was used to identify and remove high-amplitude artifacts (e.g., blinks, eye movements). The 100 ms previous and the 400 ms following each crossing of the positive amplitude threshold were removed from the analysis (enough to reject the full blink waveform). We set the threshold separately for each subject and recording device to the amplitude value corresponding to the top of the 95% confidence interval for the closed eyes period (i.e., the value that was higher than 97.5% of the samples recorded during the closed eyes periods). The obtained thresholds were validated by visual inspection of the open eyes periods. To avoid excessive trimming of the data, we did not count intervals over the amplitude threshold shorter than ten samples as blinks, and therefore we included them in the analysis. Note that, while this methodology will detect other recording artifacts that are not blinks, visual inspection of the data shows that blinks are in fact the vast majority of detected artifacts and we therefore refer to the detected artifacts as blinks elsewhere in this text.

### 2.5. Time Series Analysis

We used the metric presented by Darvishi [44] to calculate the similarity between the simultaneous recordings, segmented by the different tasks (closed eyes, open eyes, driving task; for each participant, we averaged the values of the metric for the two periods of closed and open eyes). This metric is similar to a cosine metric [45] in that it estimates similarity by measuring the cosine of the angle between two vectors of an inner product space, with a positive value indicating the vectors point in similar directions and negative values vectors in opposite directions. Values close to zero mean near-orthogonality of the signals. This metric adds to the standard cosine metric invariance against phase shifts, making it robust against residual alignment errors either due to imperfections in the alignment algorithm. This invariance also reduces the effect of time-variant misalignments caused by lost data in the RF channel.

Considering two sequences *X* = (*x_1_*,*x_2_*,…,*xn*) and *Y* = (*y_1_*,*y_2_*,…,*ym*), where generally *n* ≠ *m*. Assuming, without loss of generality, *m* ≤ *n* the Darvishi algorithm takes the following steps to measure the similarity [44] (Equations (1) to (4)):

(1) $$\mu_{y}=\;\frac{1}{m}\overset{m}{\underset{i=1}{\sum }}Y_{i,}$$

(2) $$X_{k}=Circshift\left(X,\left(-k+1\right)\right),\;\mu_{X_{k}}=\frac{1}{m}\overset{m}{\underset{i=1}{\sum }}X_{k_{i}},\;k=1,2,\ldots ,n$$

(3) $$S_{k}=\;\frac{\sum_{i=1}^{m}\left(X_{k_{i}}-\mu_{X_{k}}\right)\left(Y_{i}-\mu_{Y}\right)}{\sqrt{\sum_{i=1}^{m}{\left(X_{K_{i}}-\mu_{X_{k}}\right)}^{2}\sum_{i=1}^{m}{\left(Y_{i}-\mu_{Y}\right)}^{2}}}$$

(4) $$Sim\left(X,Y\right)=\;\underset{1\leq i\leq n}{\max}\left[S\left(i\right)\right]$$

where $\mu_{y}$ is the mean of *Y*, $X_{k}$ is the result of a circular shift operator which circulary shifts *X* by shiftsize samples, $\mu_{X_{k}}$ is the mean of $X_{k}$, $S_{k}$ is the covariance between $X_{k}$ and Y, and Sim(X,Y) is the max between the values of $S_{k}$.

We calculated baseline levels for the similarity metric to approximate the expected metric values for a pair of unrelated but spectrally similar signals. These baseline values allowed us to test the statistical significance of the similarity between the recordings (see Statistical Analysis and Results sections). We estimated the baselines by comparing the SOMNOwatch recording to a random sequence with similar spectral composition, as obtained by the use of autoregressive signal modelling techniques [46].

### 2.6. Spectral Analysis

Power spectrum estimations were performed using the Welch method [47]. For quantitative analysis, we used a Hamming window of 256 samples (1 s), with a 128 sample overlap between segments. Spectrograms were plotted using a 1024 sample (4 s) window with 512 sample overlap.

### 2.7. Signal-to-Noise Ratio Estimation

We quantified the difference in noise levels between the recording devices using an approach based on linear prediction coding, as in Kamel and Jeoti [48] (Equation (5)). 

Find *a_0_*, *a_1_*, *a_p_* such that they minimize

(5) $$e\left(n\right)=-\overset{p}{\underset{i=0}{\sum }}a_{i}x\left(n-i\right),\;a_{0}=-1,\;p=255$$

Linear prediction coding determines the coefficients of a forward linear predictor by minimizing the prediction error in the least squares sense. *p* is the order of the prediction filter polynomial, *a* = [1, *a_(1)_,* … *a_(p)_*]. *x* is the data of the signal to analyze (SOMNOwatch or MindWave).

We modelled the noise as additive and white, and used linear prediction to separate the flat spectral components from the “shaped” components and estimated the signal-to-noise ratio (SNR) on each second of the recording, and averaging the results across tasks. For the driving task, we also compared the SNRs for the first and second halves of the task, in order to detect any possible degradation of the signal-to-noise ratio during the recordings [17] (see Supplementary Materials).

### 2.8. Blink Recognition 

Wearable EEG devices are often used to generate control commands that trigger predefined actions (e.g., mouse clicks) based on easy recognizable signals (i.e., the eye blinks, [49]). Thus, blinking behavior might be used to compare the devices’ performance [32,50]. We calculated and compared the blink detection rates among devices and tasks. To recognize blinks, we used the detection algorithm described above.

### 2.9. Baseline Comparisons between Recording and Reference Sites

To exclude the possibility that the above described comparative analyses might be compromised by differences related to (a) recording sites (Fp1 when using the MindWave vs AF3 when using the SOMNOwatch), or to (b) reference sites (the ear lobe when using the MindWave vs the left mastoid when using the SOMNOwatch, Figure 1), we conducted an additional experiment. Five subjects (mean age ± SD = 23.2 ± 1.8 years; three males; no overlap with previous participants) ran a reduced experimental session, which only included the 12-minute resting state task. We performed these new comparisons with the SOMNOwatch.

### 2.10. Statistical Analyses

We tested our results using standard statistical techniques, with the alpha level set at 0.05. First, we tested the existence of a significant similarity between the recorded signals with both devices using a repeated measures 2 × 3 analysis of variance (ANOVA). The first factor was the *metric estimated*, with two levels: real (SOMNOwatch vs. MindWave), and baseline (SOMNOwatch vs. a random sequence) (see Section 2.5 for details on the calculation). The second factor was the *tasks* tested with three levels: closed eyes and open eyes conditions, and the driving task. Second, we compared estimated values of the SNR between the two recording devices and between the first and second half of the driving task, using a factorial 2 (*recording device*: SOMNOwatch vs. MindWave) × 2 (*recording period*: first 30 min vs. last 30 min of recording) repeated measures ANOVA. Third, we compared the blink detection rate between the two recording devices and among the three tasks, using a factorial 2 (*recording device*: SOMNOwatch vs. MindWave) × 3 (*tasks*: closed eyes, open eyes, and driving task) repeated measures ANOVA. In both cases, we studied the effects of both main factors and their interactions, and used a Bonferroni correction on the obtained *p*-values to control for multiple comparisons. Fourth, to estimate the reliability of EEG measurements during the two closed eyes periods for each device, we calculated the Spearman correlation coefficients. Finally, we tested the effect of the different SNRs obtained in real measures by studying the discriminability of the Berger effect (the activation of alpha waves during periods with closed eyes [51]) using two-tailed paired *t*-tests. To compare between recording and reference sites, we calculated linear regression models.

## 3. Results

### 3.1. Comparisons between Recording and Reference Sites

**Figure 3:**
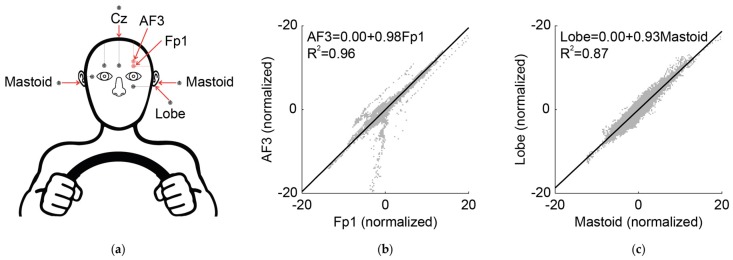
Differences between recording sites (Fp1 and AF3) and reference sites (mastoid and lobe) when recorded with the SOMNOwatch. (**a**) EEG recording configuration. Red arrows indicate the four electrodes’ placements of the SOMNOwatch used to compare the data. (**b**) Linear regression model for Fp1 and AF3 when recorded with the SOMNOwatch for five participants. The cloud of points shows the data for each subject first centered (by subtracting the average) and divided by its standard deviation, while the solid line represents the result of a linear regression of the form AF3 = b + g × Fp1. The numerical results for the regression and the correspondent determination coefficient are shown in the graph inset. (**c**) Linear regression model for Mastoid and Lobe references when recorded with the SOMNOwatch for five participants. In this case, the solid line represents the result of a linear regression of the form Lobe = b + g × Mastoid.

Data obtained using different recordings sites (*R*^2^ = 0.96; Figure 3b) and different reference sites (*R*^2^ = 0.87; Figure 3) were almost identical (for the individual participant data, see Figures S1 and S2). Therefore, in light of these complementary analyses, the possibility that the results described below might have been compromised by the different recording or reference sites seems highly unlikely.

### 3.2. Comparisons of Temporal Data Series

**Figure 4:**
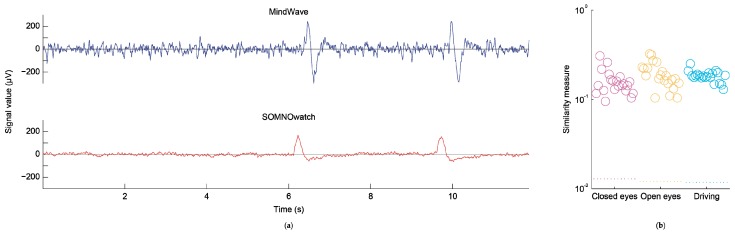
Comparison of temporal data series. (**a**) Left panel shows example traces of a simultaneous recording in one participant. The different noise levels and different shape of blinks are easy to observe. (**b**) The right panel shows the similarity measures (open circles) between the recordings for each participant and each of the three separate tasks (closed eyes, open eyes, driving task), as compared to a baseline value (dotted lines at the bottom). The values for each subject are displaced on the horizontal axis for representation purposes.

In a visual inspection of graphed data, the main differences along the time series resided in the higher noise levels found in the MindWave trace when compared to that obtained with the SOMNOwatch. Additionally, the shape of the blinks was very different, with blinks recorded with the MindWave showing a characteristic biphasic shape. The left panel of Figure 4 shows an example of simultaneous recording in a single participant. Note that while the SOMNOwatch output is calibrated to a microvolt scale, the output from the MindWave is subject to large calibration variations for each individual device, as per manufacturer specifications [52]. The right panel of Figure 4 shows the similarity metric over the recording for the three different tasks. This similarity metric is robust against small residual misalignments, such as the one observable on the left panel of Figure 4 (see Methods section). The values of the similarity metric were consistently above 0.1, indicating a positive correlation between the signals. 

The calculated baseline similarities, obtained by comparing the SOMNOwatch recordings to random sequences with similar spectral composition (see Methods section), were significantly lower than the similarities between devices, indicating an actual correlation between both recording devices. We found no significant effects of *task* in the metric, nor an effect of the interaction between the two factors (repeated measures ANOVA, effect of *metric estimated*: *F*(1,20) = 589.35, *p* < 0.05; effect of *task*: *F*(2,40) = 2.59, *p* = 0.09; first order interaction: *F*(2,40) = 3.01, *p* = 0.06).

### 3.3. Comparisons of Spectograms

**Figure 5:**
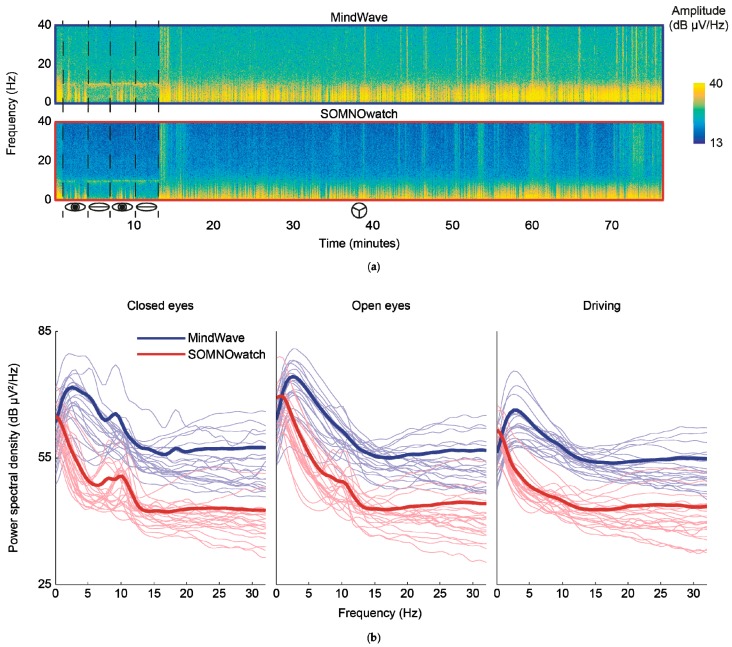
Spectral comparison between recording devices. (**a**) Spectrograms of the simultaneous recordings, in a single participant, with the two acquisition devices. The different tasks (open eyes, closed eyes, and the driving task) are delineated in the temporal axis. While the recordings are qualitatively similar, a higher level of noise can be appreciated in the MindWave data. (**b**) Power spectral densities obtained from the closed eyes (**left**), open eyes (**center**), and driving (**right**) tasks. Thin lines show individual participants, thick lines the average result. The devices differed in their response at lower frequencies, as evidenced by the MindWave peak around 3Hz.

In a visual inspection of graphed data, spectrograms from both devices were qualitatively similar, although higher noise levels can be observed in the MindWave recording. Figure 5a shows a spectral comparison between the two devices for a random single participant. We also calculated the estimated power spectral density before and after the removal of blinks and artifacts for each task, both for each of the twenty-one participants and on average (Figure 5b). As inferred from the comparison of temporal data series (Figure 4), the SOMNOwatch recordings had generally lower amplitudes. Nevertheless, the curves are fundamentally parallel for frequencies above 4 Hz. For frequencies below 4 Hz, the spectrum is quite different for both devices, with the MindWave showing a peak around 3 Hz. The recorded spectra are identical with or without blinks, except for a difference in total power due to the high amplitude of eye-related artifacts, and for blinks alone (Figure S3). The spectra are also consistent throughout the recording (Figure S4).

### 3.4. Comparisons of the Blink Detection Rate

**Figure 6:**
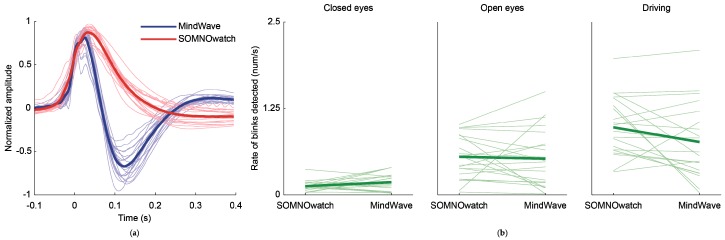
Waveform and rate of detected blinks. Average waveforms and blink detection rate for each individual participant (*N* = 21, thin lines) and the population mean (thick lines). (**a**) Average waveform, with the timepoint of crossing the amplitude threshold (see Methods section) aligned to zero. Amplitudes of individual artifacts are normalized to a maximum value of 1. The different shape of blinks is apparent. (**b**) Blink detection rate obtained from the closed eyes (**left**), open eyes (**center**), and driving (**right**) tasks. Thin lines show individual participants and thick lines are the average result.

To test if the blink detection rate was different between the two recording devices and among the three tasks, we carried out a repeated-measures ANOVA, Bonferroni corrected. The SOMNOwatch detected 6% more blinks than the MindWave, but such difference was not significant, effect of the *recording device*: *F*(1,20) = 1.14, *p* = 2.99. The effect of the *task* was significant, *F*(2,40) = 36.60, *p* < 0.001. As expected, the blink detection rate was statistically lower in the closed eyes task (mean ± SD = 0.15 ± 0.08) than in the open eyes task (mean ± SD = 0.53 ± 0.32), and both detection rates were lower than on the driving task (mean ± SD = 0.87 ± 0.41); all corrected *p*-values <0.05. The interaction between *recording device* and the *task* was also significant, *F*(1.23,24.57) = 4.10, *p* = 0.046. However, post hoc analysis of this interaction were not significant. Figure 6 illustrates the shape of the blinks recorded with both devices, as well as the advantage of the SOMNOwatch in blinking recognition. 

### 3.5. Comparisons of the Recording Quality

**Figure 7:**
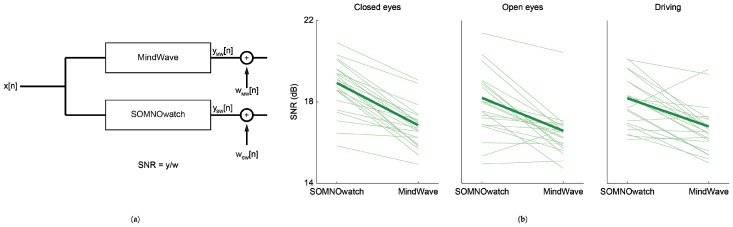
Signal-to-noise ratio (SNR) estimation. (**a**) Additive white noise model employed for the estimation of the SNR. The physiological signal *x* is filtered by the impulse response, resulting in filtered signal *y*, of both recording devices, at which point white noise (*w*) is added, resulting in the recorded signals. The SNR is defined as the ratio between the power of the filtered signal and the power of noise. (**b**) Results for each participant (thin lines) and average (thick line) the closed eyes (**left**), open eyes (**center**), and driving (**right**) tasks. SNR for the SOMNOwatch is on average 2 dB above that of the MindWave.

In order to further quantify the differences between the recordings, and specifically the effects of noise in the recorded signals, we estimated and compared the SNRs between both devices (see Methods section, Figure 7). Figure 7b shows a comparison of the estimated SNRs per each participant for both recording devices (thin lines) for each task. There was a loss of 2 dB in SNR on average between the recording devices, as denoted by the thick green line. To test if there was a degradation in recording quality, we also compared the first and second half of the driving (i.e., first 30 min vs. last 30 min of recording) task in both devices, finding no significant differences. We tested both the effect of the *recording device* and the *recording period* using a repeated-measures ANOVA, Bonferroni corrected, effect of the *recording device*: *F*(1,20) = 44.35, *p* < 0.05; effect of the *recording period*: *F*(1,20) = 0.54, *p* = 0.47; first order interaction: *F*(1,20) = 0.07, *p* = 0.79) (see Figure S4). 

**Figure 8:**
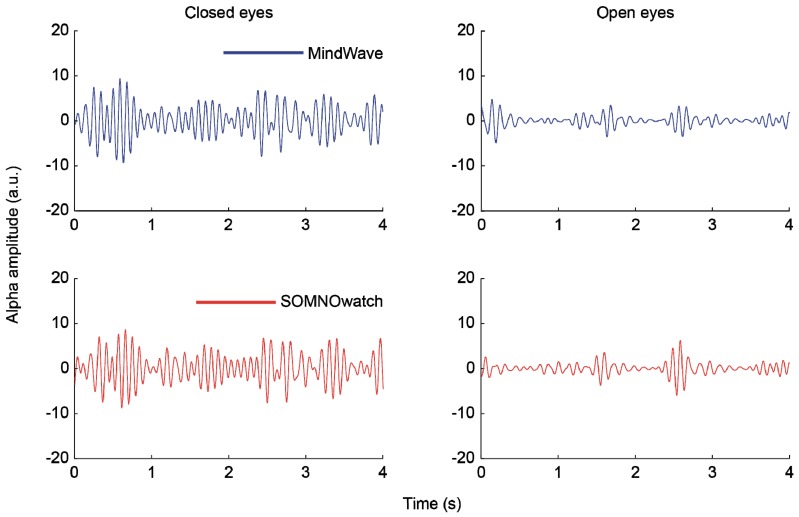
Normalized alpha waves as simultaneously captured by both devices during periods of closed eyes and open eyes. Alpha waves were separated from the rest of the signal using a 10th-order Chebychev filter. The alpha amplitude is clearly increased in closed eyes periods.

Furthermore, we analyzed how this difference in SNR affected a simple, standard EEG analysis such as differentiating between open and closed eyes states [51]. We calculated the power on the alpha band (8–12 Hz) for both periods and compared the results using a paired *t*-test. Both differences were significant. For the MindWave, *t*(17) = 2.11, *p* = 0.049, and for the SOMNOwatch, *t*(17) = 3.49, *p* = 0.002. Figure 8 shows normalized alpha waves for both devices during the periods of closed and open eyes. For a summary of results, see Table 1.

## 4. Discussion

We assessed the recording quality of the MindWave by performing simultaneous recordings with the SOMNOwatch, a traditional, medical-grade ambulatory device, and comparing the signals acquired with both devices during the performance of laboratory tasks. In a single assessment session, we recorded participants’ brain activity at Fp1 (AF3 in the case of the SOMNOwatch, see Figure 1) during a closed eyes/open eyes task and a driving simulation. We evaluated the recording quality of each device comparing the temporal data series, the spectra, the SNR, the amplitude of EEG oscillations in the alpha band, their reliability, and the blink detection rate. Whereas qualitatively the MindWave signal presents higher levels of noise and a biphasic shape of blinks, the similarity metric indicates that signals from both recording devices are indeed correlated. Moreover, the blink detection rates do not differ between the two recording devices, the amplitude of EEG oscillations in the alpha band are, as expected, different between closed eyes and open eyes for both devices, and signals coming from both devices can be considered reliable (correlating both closed eyes periods), even though reliability is lower for the MindWave. Quantitatively, the main difference between the acquired signals comes from their spectral differences at lower frequencies (<4 Hz) as well as the degradation that MindWave introduces in the SNR. 

The most salient difference is the attenuation that the MindWave device introduces at the low frequency components (<4 Hz). This power reduction is identical when considering the full recording, the recording without blinks, and the spectrum of blinks alone. This fact suggests that this is caused by the spectral (linear) properties of the combination of sensor and electronics used in the MindWave device, and not due to nonlinear effects, as would have been the case if the power reduction was different with changes in the signal amplitude, such as the ones present in blinks and artifacts. Since eye blinks have spectra with high-amplitude components into the 0.5–3 Hz band [53], we can conclude that this spectral difference is the reason for the different shape of artifacts found (Figure 6a). 

Previous validations of the MindWave include a manufacturer whitepaper in which the MindWave is compared to an unspecified Biopac device (BIOPAC Systems, Inc., Goleta, CA, USA), using an unspecified methodology [29]. This whitepaper shows the spectrum acquired with the MindWave having a similar shape to that described here, but it claims that this is due to low levels of low frequency noise and attributes it to the shorter, fixed wiring present on the MindWave. However, the manufacturing company confirmed that the results we were seeing at the low frequency components is due to a high pass filter with a cutoff frequency of 3 Hz, which is embedded in the MindWave device for controlling the low frequency noise (personal communication). Therefore, the MindWave does not actually have a better than average noise figure in low frequencies, but these frequencies are simply suppressed to avoid distortions in the waveform at the expense of lost information in this spectral band. Here, we show in a replicable manner the effect of this filter and the range of frequencies and waveform modifications it produces. Based on these findings, studies especially interested in assessing the power of the delta band (e.g., [21]) should consider that the MindWave device might not be sensitive enough to obtain reliable spectral values in these frequencies. The spectra of the recordings are otherwise similar to those obtained with the reference SOMNOwatch device, indicating good performance outside the delta band. 

Our results also show that the MindWave has a lower SNR than the reference medical-grade ambulatory device used, the SOMNOwatch. The performance of a BCI application—or any EEG configuration for research—depends on its SNR. Thus, the degradation the MindWave device introduces in the SNR is another main limitation. Researchers using the MindWave would need to plan a substantially larger number of trials [54] to counteract this lower SNR in order to obtain valid conclusions and avoid biased interpretations [55]. It is unknown whether this signal degradation occurs because of the device electronics or as a result of the limitations imposed by dry electrodes [17]. Even though dry electrodes simplify the setup procedures, are standard to many BCI applications [56] and have reached a good quality level [17], their performance is still under debate [57] as they might be more susceptible to physiological artifacts, especially due to sweat gland activity or skin stretch affecting impedance [42]. There are several possibilities to address this limitation, such as incorporating improved dry electrodes (e.g., silver plated electrodes) to the MindWave design, or to create a headset able to provide a constant force to press the electrode against the forehead.

The results of blink detection rate show that the levels of detection were similar for both devices. This result might contradict previous works about the inadequateness of the MindWave device for blink-based tasks (e.g., [32]). Differences in MindWave models; for example, the device tested by Maskeliunas and colleagues (2016) [32] had a different sampling rate (128 Hz instead of 512 Hz), might partially explain this apparent incongruence. 

Finally, to our knowledge, no previous studies of EEG comparison have been conducted in ecological or naturalistic situations, such as the one employed here (i.e., driving simulation). Thus, a comparison with the few previous works might not be straightforward. However, our results seem to expand the preliminary conclusions on the validity of this device for applied uses found on a reduced sample size (*n* = 5) [33], and to corroborate the original assessments carried out by Johnstone and colleagues [30,31]. Thus, the MindWave, while limited in terms of recording channels, might have potential value in certain EEG recording situations (e.g., [21,22]). Furthermore, our results support the preliminary observations made by [58] about the stability of the EEG signal over time in ecological everyday activities, such as a driving task.

The presented discussion must be seen in the context of three shortcomings related to the experimental methodology we used to implement the comparison: the improved overall stability of the MindWave headset, the lack of a measure of acceptability by the users, and the reduced set of compared devices. First, to facilitate the adherence between the MindWave headset and the forehead skin, we placed and secured the dry electrode with surgical tape. This solution, obtrusive and not user-friendly, might have improved the performance of the device and the quality of the recorded signals. Thus, our results should be considered in light of this technical adjustment made to ensure a constant pressure of the dry electrode against the forehead. Second, we did not provide a quantification of the overall participants’ acceptability of the investigated devices. Although our research questions were not motivated to study the final acceptance of this device, future studies should consider this subjective dimension for a holistic evaluation of the BCI tools [17]. Finally, we did focus only on one BCI device. Nowadays, several (more sophisticated and powerful) wearable EEG headsets have been introduced on the market. Whereas we focused on the MindWave for its specific features (ease-of-use and lower cost), future studies should compare the quality of the EEG signal obtained simultaneously from several devices. Although several technical issues to solve would remain (e.g., recording simultaneously from the same location with more than two devices), it is worth working in this direction. 

Overall, despite the limitations presented above, and acknowledging the need for some precautions, the MindWave has great potential that can be exploited with studies conducted in the laboratory as well as in real-world settings. Frontopolar cortex activation (in particular of the Fp1 area) is modulated by a wide range of experimental paradigms related to memory, perception (somesthesis), and motor learning [59,60]. Some practical advantages include the possibility of simultaneous recordings and a simple setup procedure for patients and other special populations (e.g., children, e.g., [23]). The stability of the recordings and the fact that the device has good linearity makes it an appropriate device for within-subjects comparisons, and for experiments in which controlled measurements are acquired with a MindWave as well (e.g., [21]). On the other hand, spectral limitations, together with the lack of precise calibration, may cause problems when comparing absolute results obtained with a MindWave with other studies in the literature, especially if comparing the power across different power bands. Additionally, the MindWave’s low signal-to-noise ratio needs to be taken into account when designing experiments and measurements employing this device, since this reduced SNR will affect the effect size and therefore statistical power.

## 5. Conclusions

Wearable EEG-based BCI devices, thanks to technological developments in dry electrodes and lowering prices, are now receiving considerable attention as potential research tools inside and outside the laboratory setting [16,17], especially in the gaming industry (e.g., [61]). Furthermore, their cost might enable a wide range of studies (e.g., involving low-income countries as well) that were not previously possible. Motivated by these considerations, we conducted a concise comparison under simple conditions that are prototypical of the basic and applied research settings in which the MindWave tends to be primarily used (e.g., [21]). The MindWave, with specific technical adjustments (see Procedure section), provides good qualitative results, and acceptable quantitative results, especially when the cost of the device is taken into account. The device is noise-limited, but provides stable recordings even over long periods of time. The results obtained are comparable to those obtained with a medical-grade ambulatory device, except for a potential calibration error and spectral differences at low frequencies. Still, since the recordings are stable, the device is valid for self-controlled experiments. 

## Figures and Tables

**Table 1:** A summary of statistical results comparing recording and reference sites (1), similarity (2), signal-to-noise ratio (SNR) (3), blink detection (4), reliability (5), and spectral analysis (6).

**1. Similarity between the recording and reference sites**	
Are Fp1 and AF3 as recording sites analogous?	Yes, *R*^2^ (AF3 = b + g × Fp1) = 0.96 *(p* < 0.001)
Are the left mastoid and the left lobe as reference sites analogous?	Yes, *R*^2^ (Lobe = b + g × Mastoid) = 0.87 (*p* < 0.001)
**2. Similarity between the recordings**	
Are similarities between devices greater than similarities between the SOMNOwatch recording and a random sequence?	Yes, *F*(1,20) = 589.35, *p* < 0.05
Do similarities between devices depend on the tasks?	No, *F*(2,40) = 2.59, *p* = 0.09
**3. SNR: Degradation in recording quality**	
Is SNR different between the two recording devices?	Yes, *F*(1,20) = 44.35, *p* < 0.05
Is SNR different between the first and the second period?	No, *F*(1,20) = 0.54, *p* = 0.47
**4. Blink detection rate**	
Does blink detection rate differ between the two recording devices?	No, *F*(1,20) = 1.14, *p* = 2.99
Does blink detection rate differ depending on the tasks?	Yes, *F*(2,40) = 36.60, *p* < 0.001
**5. Signal reliability: closed eyes periods**	
Is the EEG signal from the MindWave reliable?	Yes, *r*_s_ = 0.71
Is the EEG signal from the SOMNOwatch reliable?	Yes, *r*_s_ = 0.95
**6. Spectral analysis: Berger effect**	
Does the amplitude of EEG oscillations in the alpha band differ between open and closed eyes tasks for the MindWave?	Yes, *t*(17) = 2.11, *p* = 0.049
Does the amplitude of EEG oscillations in the alpha band differ between open and closed eyes tasks for the SOMNOwatch?	Yes, *t*(17) = 3.49, *p* = 0.002

## Supplementary Materials

The following are available online at https://www.mdpi.com/1424-8220/19/12/2808/s1, Figure S1: Differences between Fp1 and AF3 when recorded with the SOMNOwatch. Dummy in the left upper corner represents the electrode placement used. The scatter plots show samples acquired using Fp1 and AF3 on the SOMNOwatch. The data is presented for each subject individually. The cloud of points shows the individual samples, while the solid lines represent the result of a linear regression of the form AF3 = b + g × Fp1. The numerical results for the regression and the correspondent determination coefficient are shown in the graphs insets. Figure S2: Differences between references (mastoid and lobe) when recorded with the SOMNOwatch. Dummy in the left upper corner represents the electrode placement used. The scatter plots show Fp1 signals referenced to the left mastoid (A1, the reference used by the SOMNOwatch) and the ear lobe (the reference used by the MindWave). The data is for each subject individually. The cloud of points shows the individual samples, while the solid lines represent the result of a linear regression of the form Lobe = b +g × Mastoids. The numerical results for the regression and the correspondent determination coefficient are shown in the graphs insets, Figure S3: Waveform and spectra of detected blink artifacts. Average waveforms and power spectra for each individual participant (N = 21, thin lines) and the population mean (thick lines). (a) Average waveform, with the timepoint of crossing the amplitude threshold aligned to zero (see Methods section). Amplitudes of individual artifacts are normalized to a maximum value of 1. The different shape of blinks is apparent. (b) Power spectral density of detected artifacts. The shape of artifacts recorded on both devices matches that found on the full recordings, Figure S4: Spectra and signal-to-noise ratio (SNR) for the first and second half of each recording (one-hour driving task). Each period has a length of approximately 30 min. (a) Power spectral density, after blink removal, obtained with both recording devices. (b) Estimated SNRs for each participant (N = 21, thin lines) and on average (thick lines). Both measurements are stable between recordings, Table S1, Technical specifications of the MindWave and the SOMNOwatch + EEG-6 systems.
