# Supplementary material for: Validation of Electroencephalographic Recordings Obtained with a Consumer-Grade, Single Dry Electrode, Low-Cost Device: A Comparative Study
Source: Sensors (Basel). 2019 Jun 23;19(12):2808. doi: 10.3390/s19122808 (PMC6630628; doi:10.3390/s19122808)
Supplement: Supplementary file 1 [file sensors-19-02808-s001.pdf]

Supplementary material

Baseline comparisons between recording sites

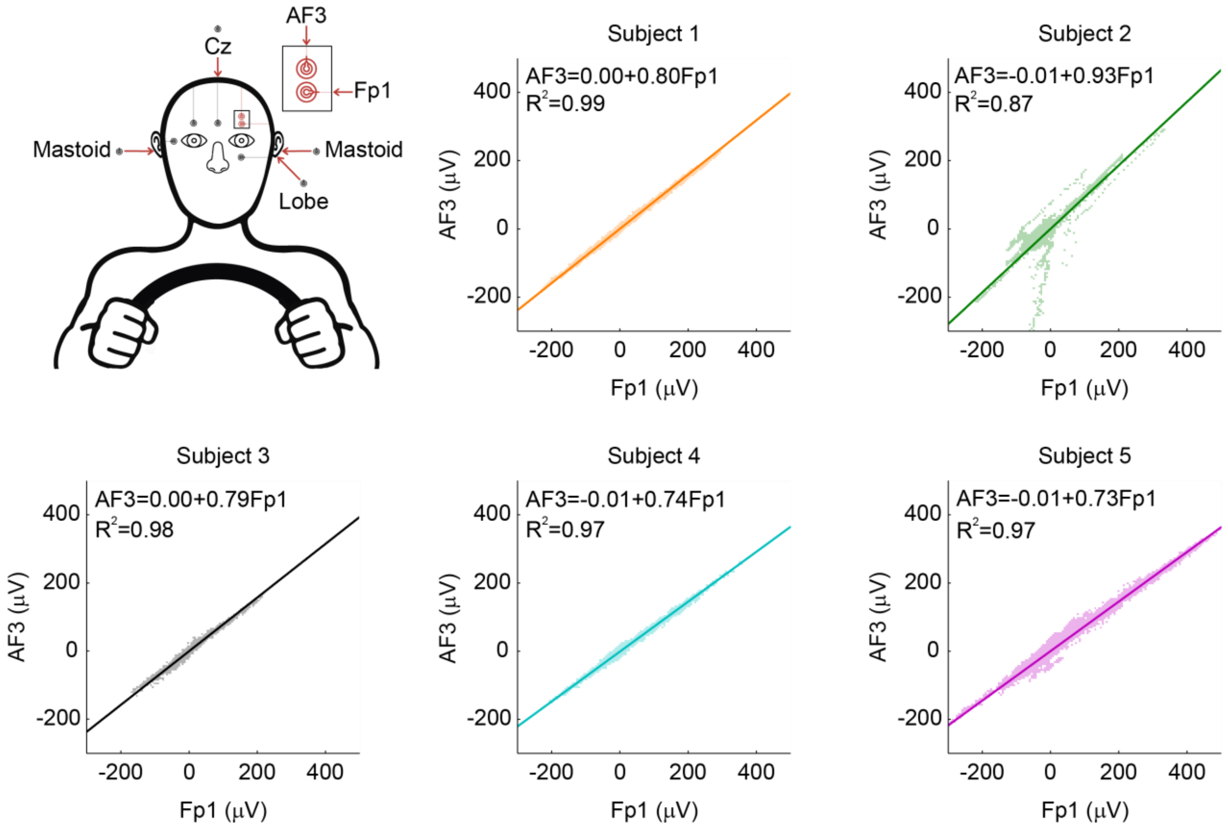

Figure S1. Differences between Fp1 and AF3 when recorded with the SOMNOwatch. Dummy in the left up corner represents the electrode placement used. Scatter plots of samples acquired using Fp1 and AF3 on the SOMNOwatch. The data is for each subject individually. The cloud of points show the individual samples, while the solid lines represent the result of a linear regression of the form AF3 = b +g*Fp1. The numerical results for the regression and the correspondent determination coefficient are shown in the graphs insets.

Baseline comparisons between reference sites

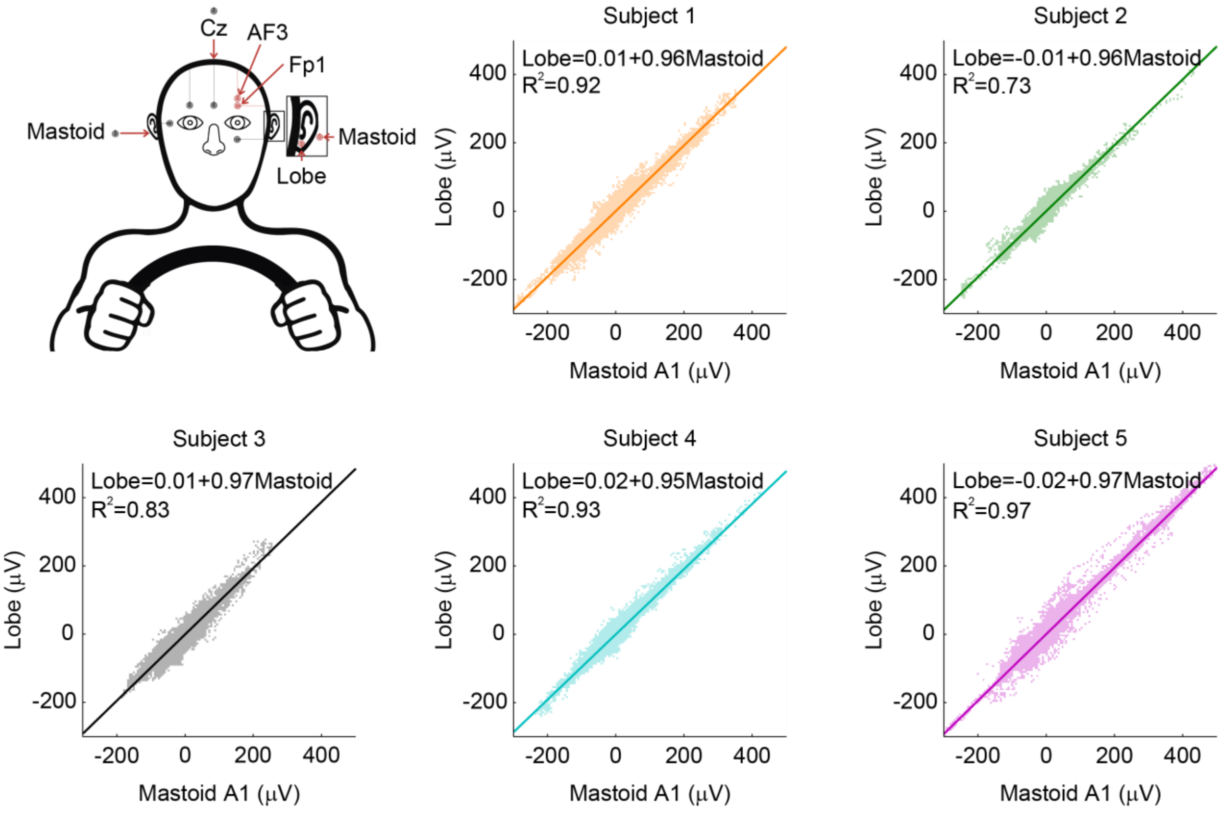

Figure S2. Differences between references (mastoid and lobe) when recorded with the SOMNOwatch. Dummy in the left up corner represents the electrode placement used. Scatter plots of Fp1 signals referenced to the left mastoid (A1, the reference used by the SOMNOwatch) and the ear lobe (the reference used by the MindWave). The data is for each subject individually. The cloud of points show the individual samples, while the solid lines represent the result of a linear regression of the form Lobe = b +g*Mastoids. The numerical results for the regression and the correspondent determination coefficient are shown in the graphs insets.

Blink artifacts waveforms and power spectra

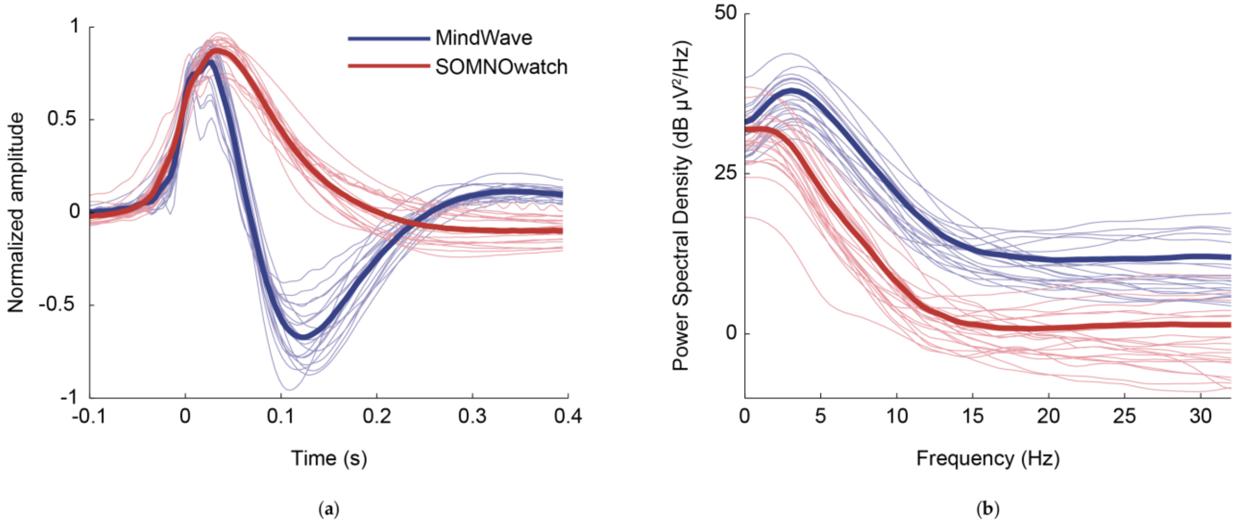

Figure S3. Waveform and spectra of detected blink artifacts. Average waveforms and power spectra for each individual participant (N = 21, thin lines) and the population mean (thick lines). (a) Average waveform, with the timepoint of crossing the amplitude threshold aligned to zero (see Methods section). Amplitudes of individual artifacts are normalized to a maximum value of 1. The different shape of blinks is apparent. (b) Power spectral density of detected artifacts. The shape of artifacts recorded on both devices matches that found on the full recordings.

Spectra and signal-to-noise ratio for the driving task

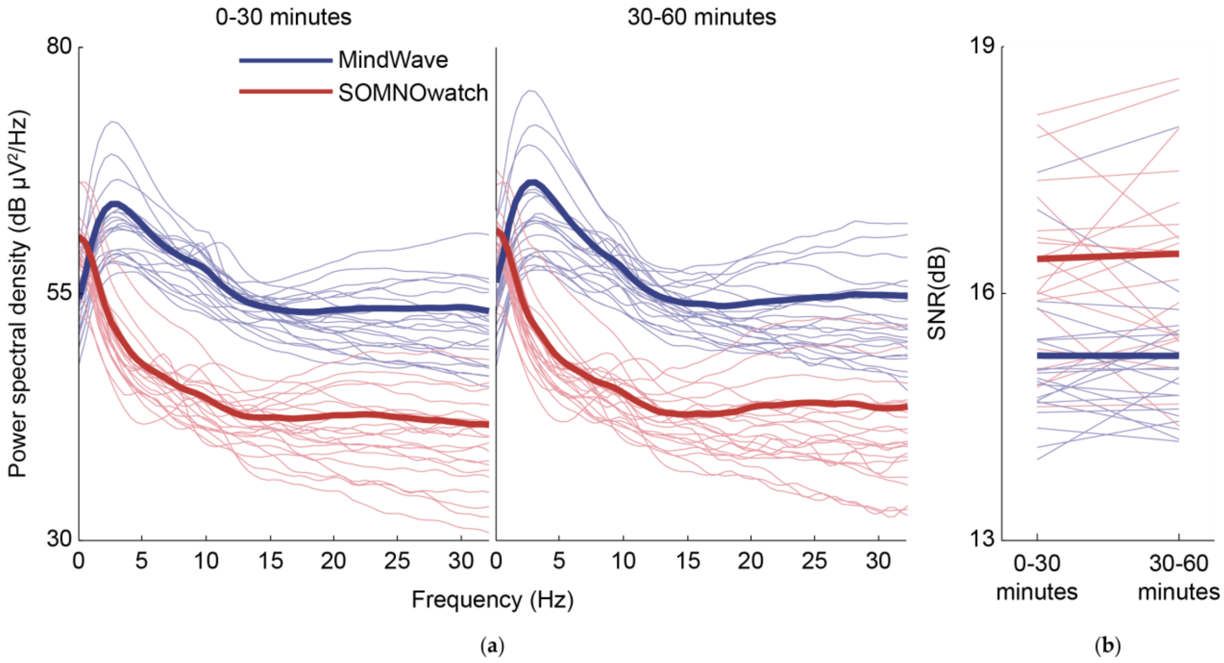

Figure S4. Spectra and signal-to-noise ratio (SNR) for the first and second half of each recording (one-hour driving task). Each period has a length of approximately 30 minutes. (a) Power spectral density, after blink removal, obtained with both recording devices. (b) Estimated SNRs for each participant (N = 21, thin lines) and on average (thick lines). Both measurements are stable between recordings.

Technical specifications

Table S1.

Technical specifications of the MindWave and the SOMNOwatch + EEG-6 systems.

System	Company, Country	Electrodes (N)	Electrodes (locations)	Electrodes (type)	Electrode (connection)	Reference locations	Sampling rate[a]	Resolution	Bandwidth	Gain	Weight	Battery Capacity
MindWave Mobile	NeuroSky Inc., USA	1	Fp1	Passive	Direct contact	A1	512Hz	12-bit ADC	3-100Hz	2000	90g	250-800mAh
SOMNOwatch + EEG-6	Somnomedics GmbH, Germany	Up to 10	10-20 system positions	Passive	Conductive gel/paste	A1/M1, A2/M2	Up to 256Hz	12-bit ADC	0.3-75Hz	2000	30g	630mAh

Note. [a] Sampling rate referred to electroencephalographic data only.

Battery life	Battery type	Measuring range	Signal quality check	Impedance check	Setup time (min)	Event marker button	Cost	SDK (Software development kit)	Software to process/analyze data	Supported platforms	Data transmission	Internal data storage	Data output
10h	AAA	±450µV	Yes	No	≈3	No	≈99$	Yes	No	Windows, Linux, Mac, iOS, Android	Bluetooth	No	Raw EEG data, two mental states (attention & meditation) and seven frequency bands[b]
Up to 50h	Li-ion	±600µV	No	Yes	≈30	Yes	≈5K$	No	DOMINO	Windows	Wired	64 MB	Raw EEG data

Note. [b] Mental states and frequency bands are calculated based on a property algorithm.
